# Research on the Application of Microcast Electromagnetic Coil in an Si MEMS Bistable Recoverable Safety and Arming Device

**DOI:** 10.3390/mi14071346

**Published:** 2023-06-30

**Authors:** Sining Lv, Hengzhen Feng, Wenzhong Lou, Chuan Xiao, Wenting Su, Wenxing Kan, Bo He

**Affiliations:** Science and Technology on Electromechanical Dynamic Control Laboratory, School of Mechatronical Engineering, Beijing Institute of Technology, Beijing 100081, China; lvsining@bit.edu.cn (S.L.);

**Keywords:** MEMS, S&A, bistable, recoverable, microcast, U-shaped electromagnetic coil

## Abstract

A low-driving energy and bistable recoverable MEMS safety and arming device (S&A), based on microcasting technology and deep silicon etching technology, is proposed to meet safety system requirements. A force–electromagnetic combination solution is constructed for the Si MEMS S&A, with parameters and strength verified, ultimately achieving an S&A size of (13 × 13 × 0.4) mm. Additionally, a low-driving energy U-shaped electromagnetic coil (USEC) model is designed using microcasting technology, and an electrical–magnetic–mechanical coupling mathematical model is established to explore the relationship between design parameters and driving capacity and reliability. With a driving power of 8 V/0.5 A, the model achieves a stable electromagnetic driving force of 15 mN with a travel distance of 0.5 mm. Finally, the fabrication and testing of the USEC and S&A are carried out, with driving capability and S&A disarming ability tests conducted to verify the feasibility of the system design. Compared to the existing S&A, this scheme has the advantages of low-driving energy, recoverability, fast response speed, and strong adaptability.

## 1. Introduction

The modern new fuses that use micro-electro mechanical systems (MEMS) technology have the advantages of low cost, mass production capability, high efficiency in design, and ease of system integration. MEMS-based miniature fuses can adapt to the limited space on the projectile, providing more space for the integration of other components, such as communication equipment, sensors, and explosive sequence control [1].

S&A is the core component of a fuse, which achieves energy transfer and isolation through a movable blast-proof mechanism. Considering the requirements for being self-disabling and self-invalidating, S&A needs to balance the functions of disarming and recoverability. The performance of S&A is key to ensuring the safety, reliability, and destructive efficiency of weapon systems. MEMS S&As are classified according to the driving principle, mainly including environmental force-driven [2,3], electrothermal-driven [4,5], electromagnetic-driven, pyrotechnic-driven [6], and other forms of driving [7,8].

The main problem of most environmental force-driven S&As are that the spring mass and inertial force are small, making it difficult to resist other disturbances such as adhesion and friction. It is also difficult to achieve the function of recoverability after disarming. The problem with electrothermal driving is that it is sensitive to temperature and requires a displacement amplification mechanism for the entire system due to the low-thermal expansion coefficient of silicon, which increases the complexity of the overall structure [9]. The problem with pyrotechnic driving is primarily safety related, and it requires higher device strength. Additionally, there are compatibility issues between pyrotechnic products and the integration of S&A. Other forms of driving, such as piezoelectric driving, require a large amount of driving energy and are difficult to integrate into the system.

Considering the simplicity, reliability, and low-driving energy requirements of the S&A, electromagnetic driving is the simpler, more reliable, and highly adaptable solution that can be used under weak environmental force conditions while being combined with advanced silicon-based manufacturing methods. The electromagnetic drive generates a magnetic field through the magnetic effect of the current, driving the explosion-proof slide block to release the safety catch. In 2010, Wu Zhiliang et al. designed an S&A driven by three cylindrical electromagnetic coils, which can achieve a drive stroke of 3 mm. The overall package size was greatly increased due to the three cylindrical electromagnetic coils installed [10]. In 2019, Wang Hailong et al. proposed an electromagnetic arming and fuzing device with state recognition features. The shortest response time of the electromagnetic arming mechanism was 3 ms. However, the driving voltage of the system was too high and the response time was relatively long [11]. In 2020, Sun Yi et al. designed a bar-shaped electromagnetic solenoid, but the electromagnetic force in the radial direction attenuated significantly, and it attenuated to less than 5 mN at a distance of about 0.5 mm [12].

In order to address the aforementioned issues, this paper proposes a bistable recoverable MEMS S&A that is suitable for operation under weak environmental forces. The substrate is parameterized and processed using Si-based microfabrication technology, and the overall size can reach (13 × 13 × 0.4) mm. Based on the S&A design standard for fuzes, weak environmental force and electromagnetic-driven multiple driving sources are adopted in the design, and both the isolation and arming stable states are non-electrical, which improves the reliability of the system. By using microcasting technology, a USEC is prepared, with a final size of (6.4 × 3.6 × 2.1) mm. The driving voltage/current is as low as 8 V/0.5 A, and a stable electromagnetic driving force of 15 mN can be achieved under a 0.5 mm stroke. Finally, tests are conducted on the arming capability of the S&A and the USEC.

## 2. Models and Methods

### 2.1. Structural Design and Parameterization of MEMS S&A

**Figure 1:**
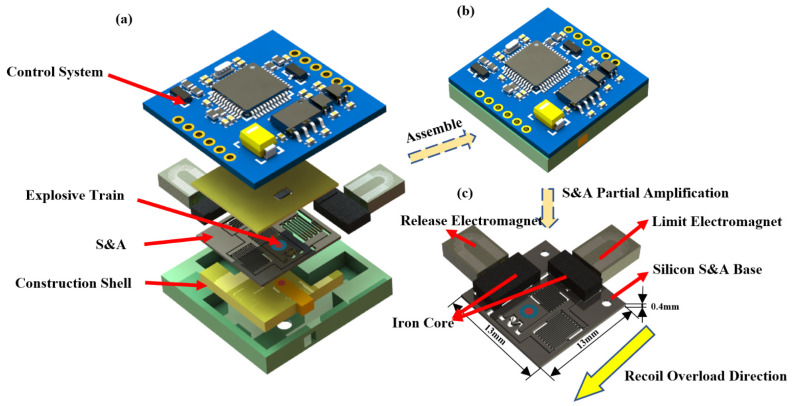
Overall structure of MEMS fuze. (**a**) Structural composition; (**b**) assembly body; (**c**) S&A detail.

The structure of the bistable recoverable MEMS S&A based on electromagnetic driving and its heterogeneous integration proposed in this paper are shown in Figure 1. The system consists of four modules: the control system, detonation sequence, structural casing, and S&A. The S&A is the core component of the system. Two safety mechanisms are integrated within an overall size of (13 × 13 × 0.4) mm, including a silicon substrate, two electromagnetic coils, and corresponding iron cores, which are used to achieve recoverable safe arming under weak environmental forces (>200 g) for the ammunition.

**Figure 2:**
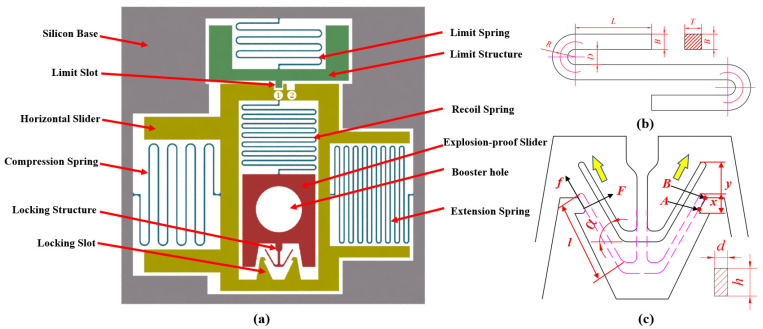
Parameterized design of the S&A. (**a**) S&A structural composition; (**b**) S-shaped spring model.; (**c**) locking mechanism model.

The S&A integrates two safety mechanisms: the recoil safety and the electromagnetic safety mechanism. The recoil safety mechanism is used to arm under weak environmental recoil forces, and the electromagnetic safety works by energizing the electromagnetic coils to generate electromagnetic forces to attract the iron core and slider to arm. The two safety mechanisms are nested together. In the unarmed state, the semiconductor bridge is aligned with the copper azide pillar, making it impossible to transfer explosive energy. When disarmed, the slider moves logically to the aligned position, aligning the semiconductor bridge, copper azide, and detonating explosive to achieve energy transfer. The overall structure is shown in Figure 2.

**Figure 3:**
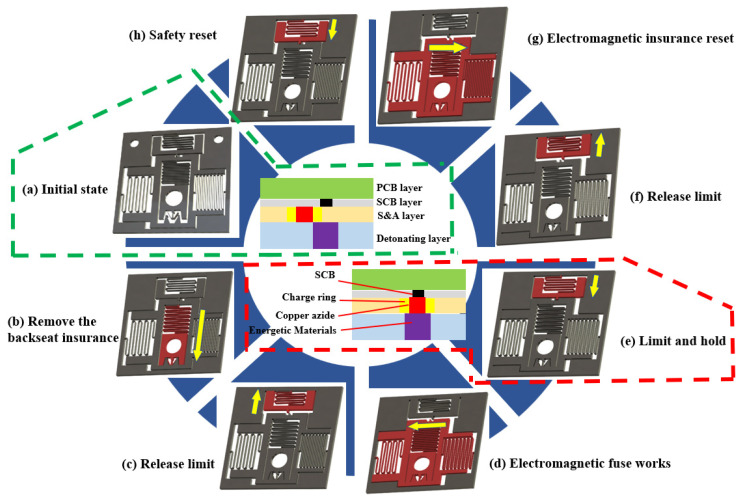
The basic principle and release process of S&A.

The explosion sequence transmission in MEMS S&A uses an “L”-shaped motion mode, and the working principle is shown in Figure 3. In the case of weak environmental forces, the recoil safety mechanism causes the explosion-proof slide block to move downward to the locking mechanism, completing the release of the first safety mechanism. After the release of the recoil safety mechanism, the limit electromagnet is energized to attract the limit module to release the limit slot 1, allowing the horizontal explosion-proof slide block to be in a free state. At this time, the MEMS release electromagnet is energized to attract the horizontal explosion-proof slide block to move to the left, overcoming the elastic force of the left and right springs. After reaching the set position, the limit electromagnet is turned off, and the limit module loses its electromagnetic force. Under the action of the MEMS spring elastic force, the limit module returns to its original length and enters limit slot 2. At this time, the release electromagnet is turned off to complete the release of the second safety, and the MEMS safety system enters a ready-to-fire state. At this time, there is no electromagnetic signal inside the system, which is a stable state. When the safety system needs to be reset, the limit electromagnet is energized to release the limit mechanism from limit slot 2, allowing the horizontal explosion-proof slide block to return to free state. Under the action of the MEMS springs on the left and right sides, the block returns to safety position.

This article uses a flat S-type spring as the main form of the spring. The spring is composed of *n* identical structural units, and the shape and basic form of each unit are shown in Figure 2b. Within a certain range of linear elasticity, the deformation of the micro-spring can be calculated using the Castigliano’s second theorem, by superimposing the small displacement $\delta_{i}=\frac{\partial U}{\partial P_{i}}$ of the micro-spring under the applied force. Here, i is the load on the micro-unit, $\delta_{i}$ is the displacement of the module structure in the direction of the applied force, U is the deformation energy of the structure, and $P_{i}$ is the i load applied to the structure. By using the partial derivative of the energy of the cantilever beam with respect to the applied force and calculating the total displacement δ at the end of the cantilever beam, the stiffness K=F/δ of the folded micro-spring can be expressed according to linear elasticity theory. The S-type micro-spring’s stiffness in the y direction can be derived [13].

(1) $$K=\frac{F}{\delta_{total}}=\frac{3EI}{n\left[4L^{3}+6{\left(D+B\right)}^{2}L+3\pi (D+B)L^{2}+3\pi {\left(D+B\right)}^{3}/8\right]}$$

where E is the elastic modulus of the micro-spring material, I is the moment of inertia of the cross-section, $I=\frac{B^{3}T}{12}$, *B* and *T* are the width and thickness of the folded cantilever beam cross-section, respectively, *L* is the length of the folded cantilever beam, *R* is the median radius at the circular bending, and *D* is the gap width of the folded beam, where *D* = 2*R* − *B*.

The explosion-proof slide block is simplified into a mass-spring-damper system, and a model is established in the direction of motion as

(2) $$m\frac{dx^{2}}{dt^{2}}+c\frac{dx}{dt}+Kx(t)=F(t)$$

where *m* is the mass of the moving part of the system, $m=\rho_{si}V_{1}$; c is the damping coefficient of the system, *K* is the equivalent stiffness, x(t) is the function of the displacement of the mass with time, F(t) is the function of the electromagnetic force over time. Meanwhile, the maximum allowable stress $F_{\max}=\varepsilon \sigma S$, ε is safety factor, σ is maximum allowable stress, and spring cross-sectional area S are considered comprehensively, S=T×B.

For the design of the locking mechanism, as shown in Figure 2c, the model of the locking mechanism can be simplified into the deflection and deformation model of a cantilever beam [14]. The stroke of the locking mechanism is *y*, the angle between the cantilever beam and the horizontal direction is α, point A is the contact point when in contact, the locking head moves downward to point B, and the movement distance is $\frac{x}{\sin\alpha }$. The length of the cantilever beam is *l*, *F* is perpendicular to the cantilever beam, f is parallel to the cantilever beam f=uF. The point where the locking mechanism receives the maximum resistance is point A, and the corresponding deflection is $w=F{\left(l-\frac{x}{\sin\alpha }\right)}^{3}/3EI$, and the maximum resistance is

(3) $$F=F\cos\alpha +\mu F\sin\alpha =\frac{3EIw}{{\left(l-\frac{x}{\sin\alpha }\right)}^{3}}(\cos\alpha +\mu \sin\alpha )$$

The point with the maximum deflection is point B, and the maximum deflection is $w=\frac{Fl^{3}}{3EI}$. W is the section modulus $W=\frac{bh^{2}}{6}$, and the corresponding strength condition is

(4) $$\sigma_{\max}=\frac{Fl}{W}=\frac{18EIw}{dh^{2}l^{2}}$$

According to the relationship between the equivalent stiffness, resistance, and electromagnetic force of the micro-spring, using the safe yield factor ε of silicon as 0.2, the design parameters of the S&A for the micro-spring and locking mechanism are obtained as shown in Table 1.

**Figure 4:**
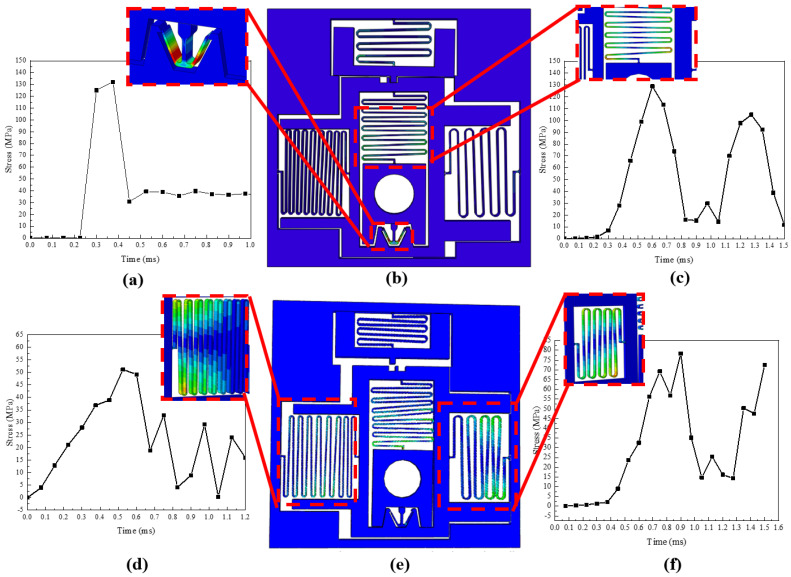
S&A dynamics simulation results. (**a**) Stress of the locking mechanism; (**b**) simulation of releasing the back−up lock; (**c**) stress of the back−up spring; (**d**) stress of the tension spring; (**e**) simulation of releasing the electromagnetic lock; (**f**) stress of the compression spring.

Based on the aforementioned design parameters, explicit dynamic simulation is performed using the ABAQUS finite element simulation software, and the stress simulation results of the key design parts, including the micro-spring and locking mechanism, are shown in Figure 4a–f. Figure 4b,e respectively show the simulated stress results of releasing the back-up and electromagnetic locks. It can be seen that the maximum stresses of the locking mechanism and spring during the release process are 130 MPa, 131 MPa, 53 MPa, and 77 MPa, all of which are less than the permitted stress of 140 MPa. Hence, no fracture will occur, and the design meets the requirements.

### 2.2. Design and Simulation of Electromagnetic Actuator

**Figure 5:**
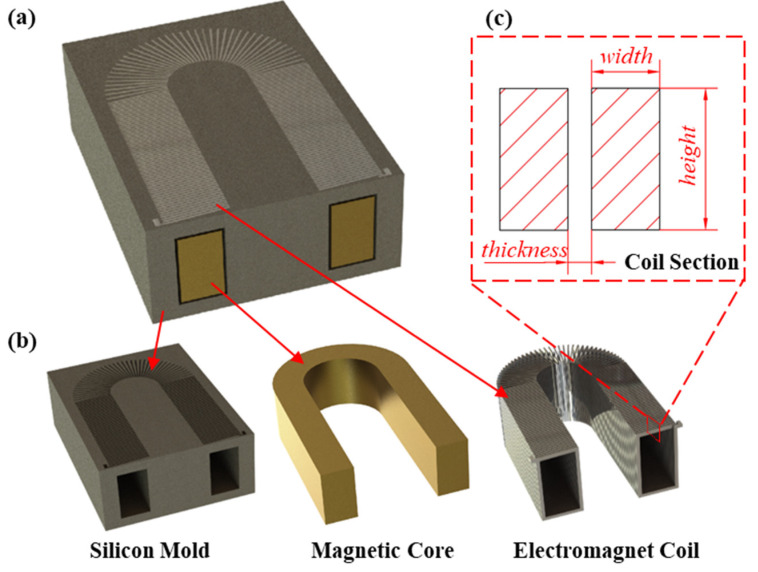
The overall structure and components of an electromagnetic coil. (**a**) Schematic diagram of the overall electromagnetic coil; (**b**) composition module of the electromagnetic coil; (**c**) cross-section of the electromagnetic coil.

The USEC designed in this paper is a chip-type coil manufactured using MEMS bulk silicon etching technology and MEMS-casting technology. The coil has 155 turns and is built on a U-shaped silicon steel core, with overall dimensions of (6.4 × 3.6 × 2.1) mm, as illustrated in Figure 5a. The electromagnetic coil comprises three components: the silicon mold, the U-shaped magnetic core, and the electromagnetic wire coil, as shown in Figure 5b. Compared to a straight coil electromagnetic coil, a U-shaped structure coil can create a closed magnetic circuit when attracting ferromagnetic substances, resulting in an electromagnetic force that can be increased several times larger than that of a straight electromagnetic coil. The working principle of the electromagnetic coil involves the magnetic core made of soft magnetic material being aligned and magnetized by the magnetic field produced by the energized coil. The energized coil generates a magnetic field with a closed magnetic circuit based on the electromagnetic induction principle. When the two magnetic fields overlap, the ferromagnetic substances near the magnetic field are attracted and displaced due to the influence of the electromagnetic force.

The coil wire material used is a Zn-Al alloy, while the magnetic core is a U-shaped core made of high permeability 1J22 alloy. This type of alloy has a high-saturation magnetic induction intensity, which results in the generation of a large attraction force at the same cross-sectional area when used as an electromagnet. Moreover, due to its high Curie point, this alloy can operate at higher temperatures than other soft magnetic materials, which have the tendency to completely demagnetize and lose magnetic stability. Additionally, its large magnetostrictive coefficient makes it highly suitable for use as a magnetostrictive transducer, with high-output energy and work efficiency. The material parameters are shown in Table 2.

A mathematical model for the electromagnetic force of an electromagnetic iron is established using Maxwell’s equations. For a static electromagnetic field, the Maxwell equations can be written as follows [15]:

(5) $$\left\{\begin{array}{l} \nabla \times H=J\\ \nabla \times E=-\frac{\nabla B}{\partial t}\\ \nabla \times B=0 \end{array}\right.$$

where *E* and *H* are the electric and magnetic fields, respectively, *J* is the current density and *B* is the magnetic induction intensity B=μH. The equations describe how the electric and magnetic fields are generated and behave in a static electromagnetic field. The attractive force of the electromagnetic iron during a steady-state operation can also be calculated based on the empirical formula

(6) $$F=\frac{B^{2}}{2\mu_{0}}S=\frac{{\left(\frac{NI}{\delta }\mu_{0}\right)}^{2}}{2\mu_{0}}S=\frac{{\left(NI\right)}^{2}\mu_{0}}{2K_{f}^{2}\delta^{2}}S$$

where *B* represents the magnetic flux density in the air gap, $\mu_{0}$ represents the permeability of vacuum, *S* represents the cross-sectional area of the magnetic circuit, *N* represents the number of turns in the coil, *I* represents the current intensity, *δ* represents the length of the air gap, and *K_f_* represents the leakage coefficient. Equation (6) shows that the electromagnetic force is directly proportional to the number of coil turns, current intensity, permeability of vacuum, and cross-sectional area, and inversely proportional to the leakage coefficient and gap distance. Figure 5c shows the cross-sectional model of the electromagnetic coil and explores the relationship between the coil width *b*, height *a*, and gap distance *c* with the electromagnetic force.

A physical model of the electromagnetic coil and S&A is established using the COMSOL Multiphysics finite element simulation software, with the material model parameters displayed in Table 3.

**Figure 6:**
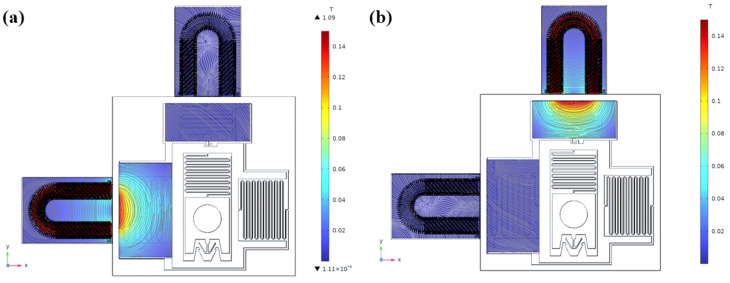
Electromagnetic simulation of the electromagnetic coil. (**a**) Decoupled electromagnetic simulation of solenoid drive; (**b**) position-limited electromagnetic simulation of solenoid drive.

Both constant current and constant voltage driving methods are employed to simulate and obtain the magnetic field strength and consequent electromagnetic force on the iron core for both the holding and limit switch electromagnetic coils when energized. The results are presented in Figure 6.

**Figure 7:**
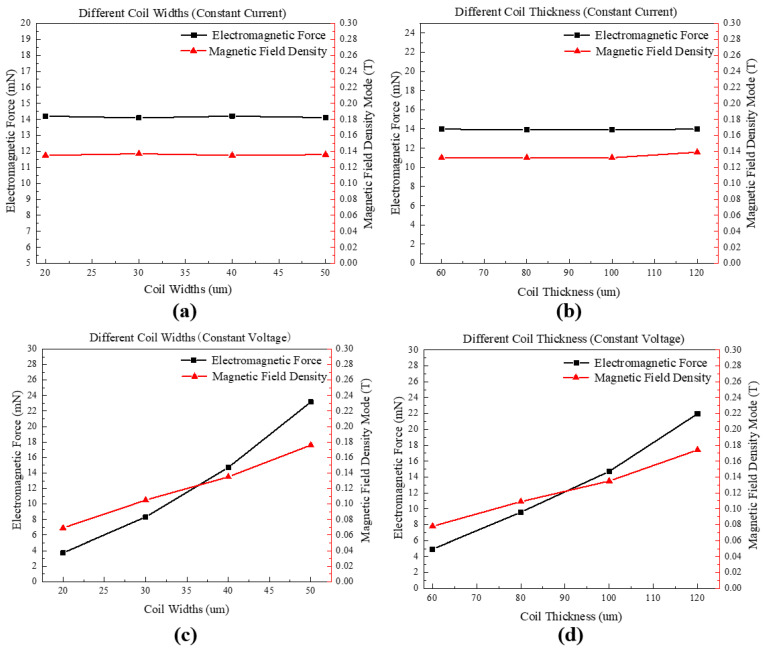
The relationship between the electromagnetic force, magnetic flux density, and coil parameters of an electromagnetic coil under constant current/voltage excitation. (**a**) Different line widths of the coil under constant current excitation; (**b**) different line heights of the coil under constant current excitation; (**c**) different line widths of the coil under constant voltage excitation; (**d**) different line heights of the coil under constant voltage excitation.

Analyses are conducted on USECs using a range of line widths (20, 30, 40, and 50 μm) and line heights (60, 80, 100, and 120 μm). Specifically, two types of analyses are carried out: (1) a constant excitation current of 0.5 A is applied, and the resulting electromagnetic force, magnetic flux density, and their relationship with the electromagnetic coil are analyzed. The results of these analyses are presented in Figure 7a,b. (2) A constant voltage excitation of 8 V is applied, and the same parameters of electromagnetic force and magnetic flux density are analyzed in relation to the electromagnetic coil. The findings for this set of experiments are presented in Figure 7c,d.

As shown in Figure 7: (1) under constant current (*I* = 0.5 A) conditions, the line width and line height of the electromagnetic coil have no effect on the electromagnetic force and magnetic flux density; (2) under constant voltage (*U* = 8 V) conditions, as the line width and line height increase, the electromagnetic force and magnetic flux density also increase accordingly. The main reason for the above phenomenon is that under the constant voltage driving mode, as the cross-sectional area increases, the resistance decreases, and the current increases, resulting in an increase in the electromagnetic force. Under the constant current driving mode, assuming that the thermal effect of the current on the resistance of the coil is not considered and a constant resistivity is adopted, the cross-sectional area will not affect the electromagnetic force.

**Figure 8:**
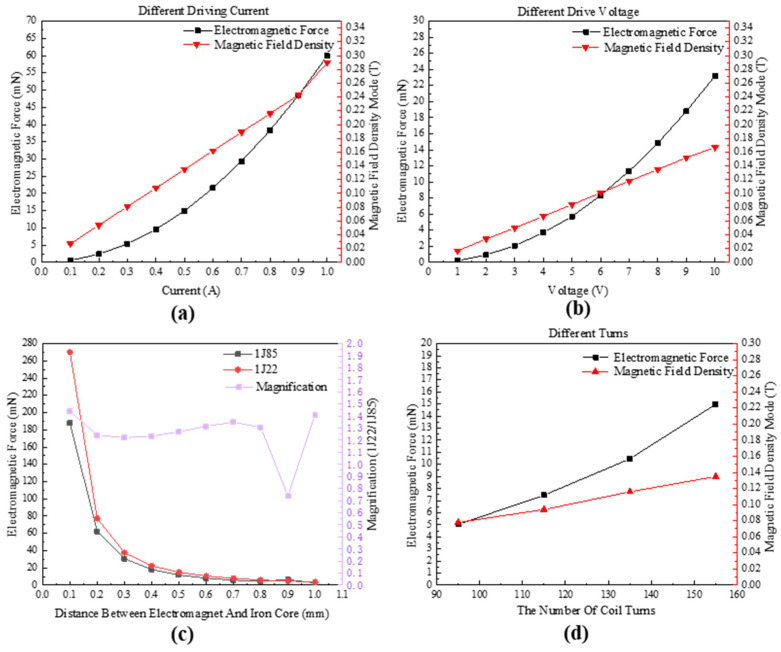
Simulation of variable parameters for USEC. (**a**) Different driving currents; (**b**) different driving voltages; (**c**) different magnetic core materials; and (**d**) different coil numbers.

This paper has established parameterized scanning models for electromagnetic actuators with variations in driving voltages, driving currents, magnetic core materials, and coil numbers. Simulations are conducted to investigate the optimal material, number of coils, and driving power supply for electromagnetic actuator design. The results of these simulations are presented in Figure 8.

As shown in Figure 8a, this paper scanned different driving currents ranging from 0.1 A to 1 A while maintaining a distance of 0.5 mm between the electromagnetic coil and the iron core. It can be observed that with an increase in current, the electromagnetic force also increases, exhibiting a quadratic parabolic relationship. Figure 8b shows that as voltage increases, the electromagnetic force also increases. Specifically, at a current of 0.5 A and voltage of 8 V, the electromagnetic force can reach approximately 15 mN, exceeding the required design value of 10 mN by 50%. This meets the usage requirements and it is recommended to use driving parameters of 0.5 A or 8 V. Different magnetic core materials are selected for the electromagnetic coil, including the iron–cobalt–vanadium soft magnetic alloy 1J22 and the nickel–iron magnetic alloy 1J85. The simulation results are shown in Figure 8c. Due to its higher saturation magnetic induction, 1J22 has a larger electromagnetic force than 1J85, with an amplification factor of approximately 1.2–1.4 times. Finally, simulations are conducted for different coil numbers, and the results are shown in Figure 8d. It can be observed that the electromagnetic force becomes more significant as the number of coils increases. The final parameters for the USEC are shown in Table 4.

**Figure 9:**
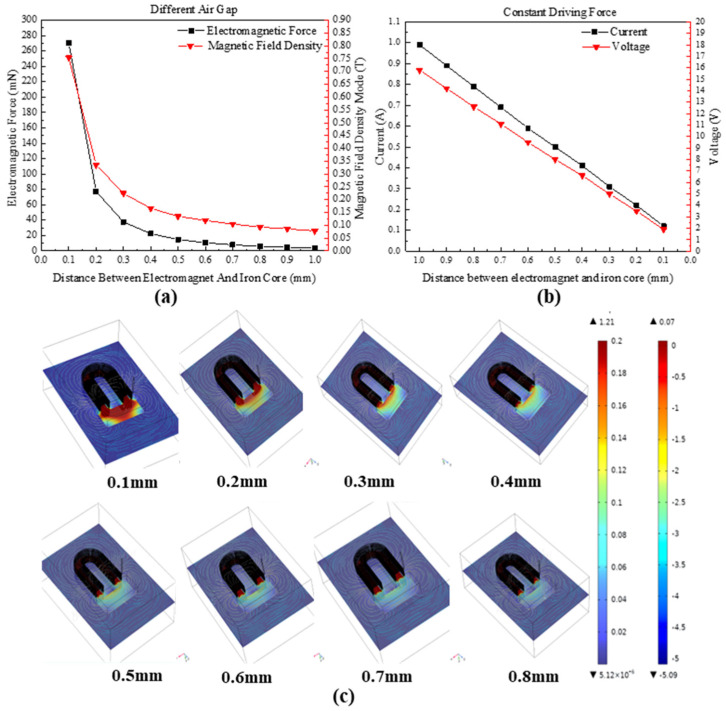
The relationship between the electromagnetic force of an electromagnet and the air gap. (**a**) Relationship between electromagnetic force and air gap distance, (**b**) changes in driving current and voltage under a constant electromagnetic force; (**c**) finite element simulations of the relationship between electromagnetic force and air gap distance.

The changes in electromagnetic force of an electromagnet under different air gap conditions are investigated, and the results are shown in Figure 9a,c. By simulating the variation in the electromagnetic force with the distance between the electromagnet and the iron core, the article reveals that, at a constant driving current of 0.5 A, the electromagnetic force rapidly decreases as the air gap increases, showing a parabolic relationship. When the air gap is 0.1 mm, the electromagnetic force can reach 270 mN, but when the air gap increases to 0.5 mm, the electromagnetic force drops significantly to 15 mN. The simulated magnetic flux density distribution is shown in Figure 9c. To reduce the power consumption of the electromagnet, a variable power driving method is considered to maintain a constant electromagnetic force of 15 mN. The results of the driving current and voltage are shown in Figure 9b. As the air gap decreases, the current and voltage decrease linearly, thus reducing the power consumption of the electromagnet.

In summary, in order to increase the attraction force of an electromagnet coil, while keeping the voltage constant, increasing the number of turns of the coil, reducing the resistance of the winding, or reducing the iron core air gap can increase the attraction force. Similarly, the magnetic circuit structure can be optimized to reduce the leakage coefficient of the magnetic circuit.

## 3. Fabrication

### 3.1. Preparation of Electromagnetic Coils

**Figure 10:**
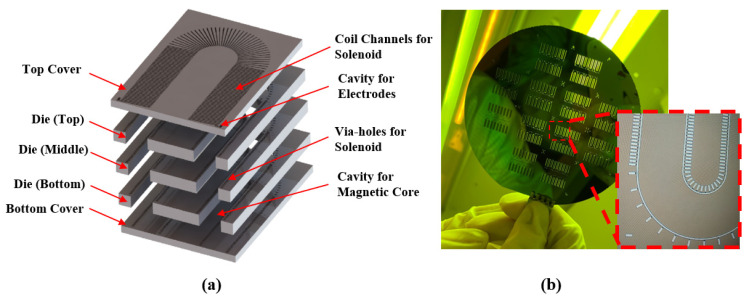
USEC structure. (**a**) Five-layer structure of USEC; (**b**) machined object.

The electromagnetic iron designed in this paper has a depth–width ratio of 1:11, and is divided into three parts: silicon mold, U-shaped magnetic core, and electromagnetic coil. The silicon mold is composed of five layers of silicon wafers, including 300 μm double-polished silicon wafers for the top and bottom layers, and 500 μm double-polished silicon wafers for the three middle layers. The five layers of wafers are stacked together to achieve a total thickness of 2.1 mm, and the overall structure is shown in Figure 10a.

**Figure 11:**
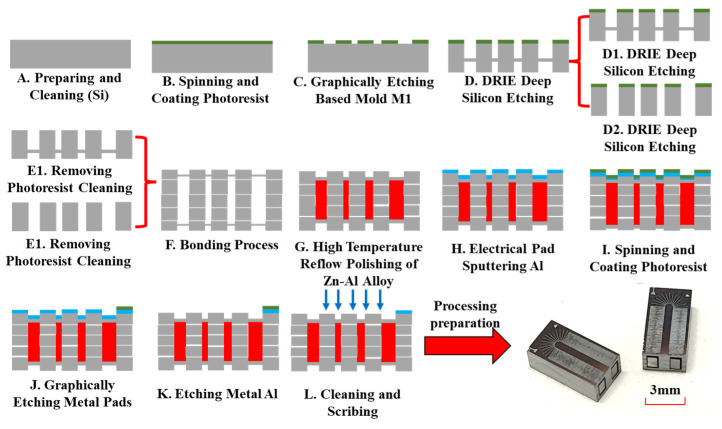
Process of MEMS casting.

Firstly, a micro-mold of electromagnetic iron is fabricated by a bulk micromachining method in silicon material. The top and bottom layers are etched by a deep silicon etching process to expose the outer parts of the coil, and the three middle layers are etched by a deep silicon etching process to create vertical through holes in the vertical direction. The electromagnetic iron micro-mold consists of surface channels and through holes connecting the upper and lower channels. The electromagnetic coil is fabricated by injecting liquid alloy from an electrode cavity into the micro-mold. The flow of liquid alloy in the helix mold is similar to a winding process, passing through the three middle layers aligned with the top and bottom layers to form a complete mold [16]. The detailed process is shown in Figure 11.

Step A: Clean the silicon-based (100) oriented substrate.

Step B and C: Spin-coat photoresist and complete the morphology of the silicon-based mold, using the M1 mask photolithography process.

Step D and E: Use the deep silicon etching process to release the structure of the electromagnetic coil, using a segmented and multi-stage etching process.

Step F: Perform plasma bonding between silicon wafers.

Step G: Cast the Zn-Al alloy into the electromagnetic coil mold using a high-temperature reflow process.

Step H–K: Prepare the electrode interface of the USEC to achieve graphical surface of the metal solder pad.

### 3.2. Process and Preparation of S&A

**Figure 12:**
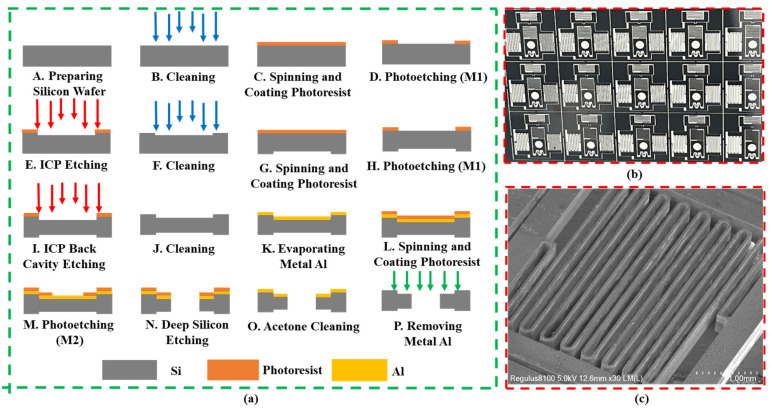
MEMS S&A deep silicon etching process (**a**) Deep silicon etching process; (**b**) Preparation of physical objects; (**c**) The photomicrographs of the silicon trench.

Regarding the MEMS S&A designed in this paper, the specific processing procedure is designed as shown in Figure 12.

Step A and B: Prepare a silicon wafer with a thickness of 400 μm and a (100) crystal orientation. Place the silicon wafer into the cleaning chamber. 

Step C and D: Coat the photoresist evenly and form a graphic pattern to create a security system frame (M1) on the front side.

Step E: Dry etching using DRIE deep silicon etching process with an aspect ratio of 1:15 and a etching depth of 30 μm.

Step F–H: Coat the photoresist evenly and form a graphic pattern to create a security system frame (M1) on the back side.

Step I: Dry etching and adopt ICP deep silicon etching technology to complete the graphic patterning of the MEMS secure system back chamber frame.

Step J and K: Clean the silicon wafer and evaporate metal Al as a metal mask.

Step L–N: Use photoresist as the mask and perform a spin-coating operation. Subsequently, proceed with the etching of the metal mask, aluminum (Al), to form a complete mask structure. Then deep silicon etching enables complete etching of the silicon wafer structure.

Step O and P: Clean the structure and remove the metal Al mask to complete device scribing. Subsequently, form a silicon-based MEMS S&A preparation.

## 4. Test and Discussion

### 4.1. Performance Testing of Electromagnetic Coil Driving Capability

In order to verify the driving ability and physical characteristics of the USEC, the resistance performance test and the thermal effect are tested through the micro-mechanical test platform. The internal resistance of 20 USECs is tested, and the internal resistance obtained is shown in Table 5.

**Figure 13:**
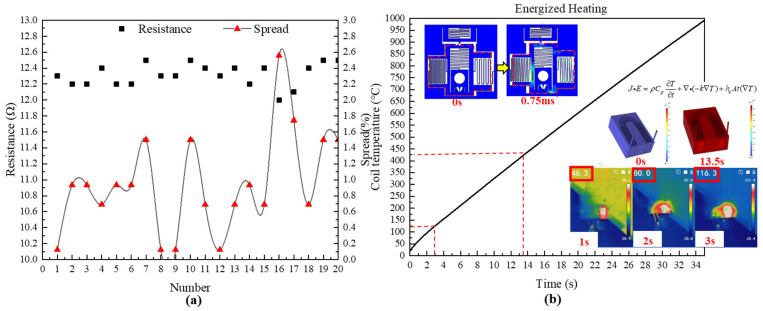
USEC resistance and temperature testing. (**a**) Resistance distribution; (**b**) simulation and testing of thermal effects.

The resistance dispersion of USEC is shown in Figure 13a. The test shows that the resistance range of the MEMS electromagnetic coil is 12.2~12.5 Ω, and the resistance spreads 2.6%, with good consistency. The simulation test of the electric heating effect is shown in Figure 13b [17]. The release time of the electromagnetic insurance is 0.75 ms according to the simulation. In order to ensure the reliability of the protection, the power-on time is set at 3 s. The temperature of the USEC is tested by an infrared thermometer, and the actual results and simulation results are compared in Figure 13b. After 3 s of power on, the temperature of the electromagnet can reach 116.3 °C, which is lower than the melting point of the Zn-Al alloy of the electromagnetic coil at 420 °C.

**Figure 14:**
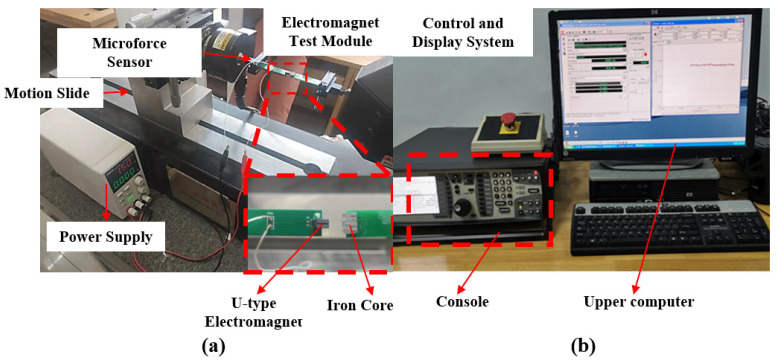
Microelectromechanical testing system. (**a**) Micro-mechanical testing platform; (**b**) host computer control and display system.

In order to test the electromagnetic force driving performance of the electromagnetic coil, the micro-mechanical testing system is used to analyze the relationship between the USEC of electromagnetic force and displacement. The testing accuracy of the instrument is 1 mN, and the experimental system diagram is shown in Figure 14.

**Figure 15:**
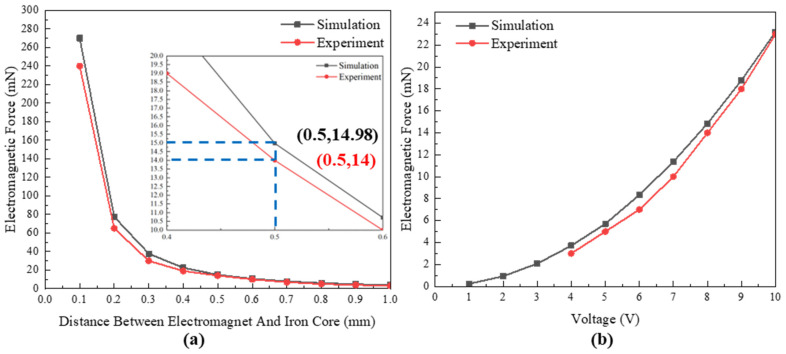
Testing of electromagnetic driving force. (**a**) The relationship between electromagnetic force and distance; (**b**) the relationship between electromagnetic force and driving voltage.

The USEC is fixed on the circuit board, and the electrodes are connected to the power supply via wires. One end of the circuit board is clamped, while the magnet IJ22 is fixed on the other end, with the computer-controlled console used to adjust its cross-section to face the cross-section of the electromagnetic coil exactly, and the distance between the magnet and the coil can be adjusted with precision to the level of micrometers. Meanwhile, a force sensor is connected to the computer system, and an image of the electromagnetic force and displacement can be obtained based on the distance between the electromagnetic coil and the magnet. The results are shown in Figure 15.

From the experimental results, it can be concluded that under a constant driving voltage of 8 V, the electromagnetic force rapidly decays as the distance between the electromagnet and the iron core increases; when the spacing is 0.1 mm, the electromagnetic force can reach 240 mN; when the spacing increases to 0.5 mm, the electromagnetic force is approximately 14 mN as shown in Figure 15a. As the driving voltage/current increases, the electromagnetic force induced by the electromagnetic coil also gradually increases. When the voltage range is 0~10 V, the electromagnetic force range is 0~23 mN, as shown in Figure 15b. The result satisfies the requirement of the safety system release force.

### 4.2. S&A Electromagnetic Release Test

**Figure 16:**
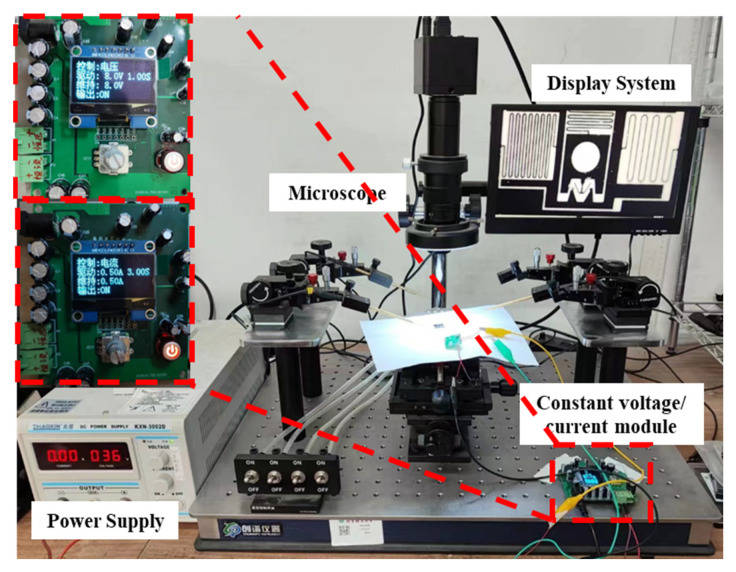
Electromagnetic-driven S&A testing platform.

The system-level integration and experimental validation of S&A are performed, with the testing platform shown in Figure 16. Following the system flow and driving logic, the USEC is powered sequentially, and the iron core fixed on S&A drives the compression and stretching of the planar spring on it, achieving the motion of critical components such as the slider and limit mechanism.

**Figure 17:**
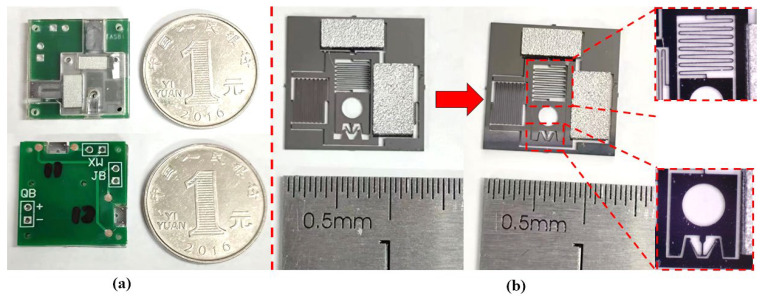
Heterogeneous integration of microsystems and release of recoil safety mechanism. (**a**) Integrated dimensions of fuze microsystems; (**b**) simulated release of recoil safety mechanism.

The final state of the S&A integration designed in this article is shown in Figure 17a. The simulated release of the recoil safety mechanism is shown in Figure 17b, where both the MEMS plane spring and the locking mechanism can achieve normal movement.

**Figure 18:**
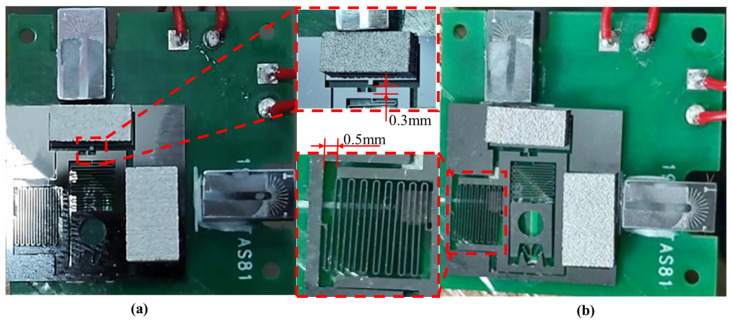
The process of electromagnetic insurance release. (**a**) Slider limit release; (**b**) electromagnetic safety release.

The electromagnetic-driven release test is conducted on MEMS S&A, and the results obtained are shown in Figure 18. Under the action of electromagnetic force, the upward movement stroke of the limit mechanism reaches 0.3 mm. The limit mechanism disengages from the limit groove and ultimately releases the limit on the slider, as shown in Figure 18a. Under the action of the electromagnetic coil in the horizontal direction, the slider moves 0.5 mm to the right to release the electromagnetic safety and realize the alignment of the slider, as shown in Figure 18b. It is proven that the design function can be realized.

The main subjects of comparison in this paper are those that adopt low-voltage/low-current electric heating drive forms. In addition, electromagnetic driving is classified into three types: electromagnetic locking, electromagnetic attraction, and electromagnetic induction. The main technical parameters of different schemes are compared as shown in Table 6.

From Table 6, it can be seen that the electromagnetic driving mode designed by Wu et al. [10] has a large displacement but a longitudinally large size. Lv et al.’s [18] electromagnetic induction safety system has too little of a displacement, while Sun Yi et al.’s [12] electromagnetic locking safety system cannot achieve bistability. On the other hand, electromagnetic driving has certain advantages in driving displacement, scale, and energy, but it is difficult to pass the temperature shock test of the safety system and is easily affected by external temperature. Taking into account the factors of size, driving energy, and integration, the integration approach of USEC and silicon-based S&A employed in this study offers the benefits of compact size and lower driving power, enabling a bistable recoverable operation under weak environmental force, and thus presenting a high level of practicality.

## 5. Conclusions

In this study, a novel bistable recoverable MEMS switch and actuator (S&A) that is adaptive to weak environmental forces is proposed. The device, which is small in size, low in power consumption, and designed with integration in mind, demonstrates an effective improvement in the system’s reliability and recoverability. The S&A utilizes micromachining technology to create a USEC as a driving force and a flat spring and slider to switch quickly from isolation to a ready state. Furthermore, the S&A substrate is parameterized and designed using silicon-based micromachining, with dimensions of (13 × 13 × 0.4) mm. A low-energy driving electromagnetic coil model is designed, and a USEC is manufactured using the micromachining process. Additionally, a mathematical model of electrical–mechanical force coupling is established to optimize the design. The tests show that a low-driving voltage of 8 V can generate a stable electromagnetic driving force of 15 mN within a 0.5 mm travel distance, and the system is able to achieve two-stage insurance release and a steady-state recoverable state. In summary, the USEC-based S&A designed in this study achieves a new form of electromagnetic safety and arming system. The designed S&A system enhances the intelligence, safety, and adaptability of the device compared to previous research, leading to higher functional integration, which possesses high practical value.

## Figures and Tables

**Table 1:** S&A design parameters.

Type	Value	Type	Value
$\rho_{si}$ (kg/m^3^)	2330	*R* (μm)	100
*E* (GPa)	112.4	*L* (μm)	1200
*σ* (MPa)	700	*h* (μm)	400
*ε*	0.2	*d* (μm)	60
*B* (μm)	60	*l* (μm)	800
*T* (μm)	400	*α*	60°

**Table 2:** The parameters of 1J22 alloy.

Alloy	Resistivity (μΩ·m)	Density (g/cm^3^)	Curie Point (°C)	Saturation Magnetostriction Coefficient (×10^−6^)	Elastic Modulus (GPa)	Saturation Magnetic Induction (T)
1J22	0.4	8.2	980	60~100	216	2.4

**Table 3:** Simulation material parameters for the electromagnetic coil.

Parameters	Si	Zn-Al Alloy	IJ22 Alloy
Conductivity (S/m)	1 × 10^−12^	1.5 × 10^7^	14.6
Relative permittivity	11.7	1	1
Saturation magnetic flux (T)	-	-	2.4
Resistivity (μΩ·m)	-	-	0.4

**Table 4:** The final parameters for the USEC.

Width (μm)	Thickness (μm)	Coil Numbers	Current (A)	Voltage (V)	Core Material
40	100	155	0.5	8	1J22

**Table 5:** The internal resistance of the USECs.

**Serial Number**	**#1**	**#2**	**#3**	**#4**	**#5**	**#6**	**#7**	**#8**	**#9**	**#10**
Resistance (Ω)	12.3	12.2	12.2	12.4	12.2	12.3	12.5	12.3	12.3	12.5
**Serial Number**	**#11**	**#12**	**#13**	**#14**	**#15**	**#16**	**#17**	**#18**	**#19**	**#20**
Resistance (Ω)	12.4	12.3	12.4	12.2	12.4	12.0	12.1	12.4	12.5	12.5

**Table 6:** Comparison of existing studies.

Year	Authors	Driving Mechanism	Maximum Output Displacement	Material	Device Size (mm)	Drive Parameters
2010	Wu et al. [10]	Electromagnetism Attraction	3 mm	Ni	13 × 13 × 20	10 V
2015	Lv et al. [18]	Electromagnetism Induction	55 μm	Si	-	8 mA
2019	Hu et al. [19]	Electro-thermal	2 mm	Si	8.5 × 8.5 × 0.8 (only S&A)	11 V
2020	Sun Yi et al. [12]	Electromagnetism Locking	200 μm	Si	15 × 9 × 0.5 (only S&A)	5 V
2022	Wang et al. [4]	Electro-thermal	371.9 μm	Si	13.4 × 8.5 × 5.2	10 V
2023	This paper	Electromagnetism Attraction	500 μm	Si	13 × 13 × 0.4 (only S&A) 20 × 20 × 3.4	8 V/0.5 A

## Data Availability

The data presented in this study are available on request from the corresponding author.
